# Induction of *in vitro* Metabolic Zonation in Primary Hepatocytes Requires Both Near-Physiological Oxygen Concentration and Flux

**DOI:** 10.3389/fbioe.2020.00524

**Published:** 2020-06-03

**Authors:** Benedikt Scheidecker, Marie Shinohara, Masahiro Sugimoto, Mathieu Danoy, Masaki Nishikawa, Yasuyuki Sakai

**Affiliations:** ^1^Department of Chemical System Engineering, University of Tokyo, Tokyo, Japan; ^2^Department of Mechanical and Biofunctional Systems, Institute of Industrial Science, The University of Tokyo, Tokyo, Japan; ^3^Institute for Advanced Biosciences, Keio University, Yamagata, Japan; ^4^CNRS UMI 2820, LIMMS, University of Tokyo, Tokyo, Japan

**Keywords:** hepatocyte, zonation, oxygen supply, metabolic reprogramming, ADME-Tox, PDMS

## Abstract

Pre-clinical drug screening is an important step in assessing the metabolic effects and hepatic toxicity of new pharmaceutical compounds. However, due to the complexity of the liver microarchitecture, simplified *in vitro* models do not adequately reflect *in vivo* situations. Especially spatial heterogeneity, known as metabolic zonation, is often lost due to limitations introduced by typical culture conditions. By culturing primary rat hepatocytes in varied ambient oxygen levels on either gas-permeable or non-permeable culture plates, we highlight the importance of biomimetic oxygen supply for the targeted induction of zonation-like phenotypes. Resulting cellular profiles illustrate the effect of pericellular oxygen concentration and consumption rates on hepatic functionality in terms of zone-specific metabolism and β-catenin signaling. We show that modulation of ambient oxygen tension can partially induce metabolic zonation *in vitro* when considering high supply rates, leading to *in vivo*-like drug metabolism. However, when oxygen supply is limited, similar modulation instead triggers an ischemic reprogramming, resembling metabolic profiles of hepatocellular carcinoma and increasing susceptibility toward drug-induced injury. Application of this knowledge will allow for the development of more accurate drug screening models to better identify adverse effects in hepatic drug metabolism.

## Introduction

The development of pharmaceuticals requires strict compliance with regulatory requirements. Especially efficacy and toxicity of drugs are essential parameters that need to be considered before further testing in clinical trials. In these pre-clinical studies, the safety of pharmaceutical compounds is assessed through a myriad of *in vivo* and *in vitro* assays regarding their absorption, distribution, metabolism, excretion, and toxicity (ADME-Tox). Even though these functions require the interaction of multiple organs, the focus of research often centers on the liver as the main site of metabolism. While *in vivo* models do represent the metabolism of an organism adequately, utilization of animal models faces multiple issues ranging from the ethical questions regarding animal use to the applicability of results due to interspecies differences (Begley and Ellis, 2012). These problems, however, can be circumvented by simplified models of human hepatocytes cultured *in vitro*. Nevertheless, these *in vitro* models generally do not deliver the required level of accuracy due to the complexity of the hepatic metabolism and microarchitecture. For these assays to be more reliable, it is therefore imperative to develop and use highly functional and representative models of liver metabolism.

The metabolism of vertebrates consists of a multitude of different pathways, which fundamentally rely on the liver as the central organ for metabolic functions. Blood glucose homeostasis, metabolism of xenobiotics and endogenous byproducts, and synthesis of bile acids are just a few of its core functions (Kietzmann, 2017). Managing these pathways requires distinct patterning of enzymes in order to be efficient since certain pathways are inherently opposing each other. This compartmentalization is referred to as metabolic zonation, which describes the spatial difference of cellular functions along the sinusoids of the liver's structural units—the lobule. Accordingly, periportal hepatocytes, named after their location near the portal triad at the perimeter of the lobule, can be phenotypically distinguished in terms of their metabolic functions from pericentral hepatocytes located close to the central vein. This is especially relevant in terms of drug-metabolizing enzymes, which are often expressed with pericentral bias (Braeuning et al., 2006; Hailfinger et al., 2006). Although this systematic patterning is clearly described through extensive research (Halpern et al., 2017, 2018; Ben-Moshe et al., 2019), the underlying mechanisms are still not completely understood. A combination of multiple signaling pathways is alleged to be involved in the formation of this zonation phenomenon. Historically (Jungermann and Sasse, 1978), metabolic zonation has solely been associated with the unique oxygen gradient along the liver sinusoids. As blood is supplied from both the portal venule and the hepatic artery, oxygen partial pressure of entering blood is reported (Kietzmann, 2017) to be around 65 mmHg (8.5%). Due to cellular respiration, oxygen concentration then drops along the sinusoid to roughly 30 mmHg (4%) near the central vein (Kietzmann, 2017). As a result of oxygen's cellular signaling function through, resident cells then assume position-specific roles in terms of their metabolic functions. Additionally, recent research has implicated Wnt/β-catenin signaling as a key component in the modulation of metabolic zonation (Benhamouche et al., 2006; Burke and Tosh, 2006). Wnt proteins convey paracrine signaling through the activation of intracellular β-catenin, a central player in many developmental pathways (Burke et al., 2018) and, relevant in this context, pericentral gene profiles (Preziosi et al., 2018). Oxygen-dependent degradation of β-catenin ensures the specific compartmentalization is locally confined to pericentral regions. While this illustrates the interaction of multiple pathways in the zonation of hepatic tissue, it also highlights the central role of pericellular oxygen tension. With oxygen being a key regulator of tissue functionality *in vivo*, it is important to accurately model these conditions *in vitro*, especially in hepatocyte cultures due to their high metabolic activity and resulting oxygen consumption rate (OCR) (Place et al., 2017). However, limited gas diffusion through aqueous medium limits oxygen supplementation and therefore the maximal possible OCR of cells in conventional culture systems (Place et al., 2017), leading to modified systems with reduced cell density or hyperoxic environments (Kidambi et al., 2009). Although it is safe to assume that this affects metabolic functions in hepatic phenotypes, modulation of oxygen tension and flux are seldom considered together in hepatocyte-based clinical *in vitro* assays. Previous research has shown that changes in ambient oxygen concentration influence energy-related gene expression of hepatocyte cultures (Kietzmann et al., 1996), as well as the expression of drug-metabolic enzymes (Allen et al., 2005) according to zonal profiles. However, these conventional approaches generally do not allow for long-term cultures due to the limited lifetime of terminally differentiated hepatocytes (Godoy et al., 2016), especially when subjected to unnatural oxygen conditions (Guo et al., 2017). In contrast, increased oxygen flux in gas-permeable tissue culture plates, e.g., made from polydimethylsiloxane (PDMS) or fluorocarbon-polymers, has shown to greatly improve hepatocellular functionality and viability (Xiao et al., 2014), allowing for prolonged culture times.

Modulating ambient oxygen tension at biomimetic supply rates should thus allow for highly functional and viable cultures that assume zonation-like phenotypes due to zone-specific pericellular oxygen levels. Accordingly, we aim to induce stable, zone-specific hepatocytes in PDMS tissue culture plates by subjecting them to different ambient oxygen tension. In order to investigate the effect of limited oxygen supply and the resulting reduced OCR, we further compare these cultures to conventional oxygen supply conditions by blocking the gas-permeable membrane—thus limiting supplementation to diffusion through the culture medium. Understanding metabolic plasticity of hepatocytes caused by oxygen will ultimately lead to improved hepatocellular *in vitro* assays, mimicking the *in vivo* metabolism more accurately and thus allowing for more relevant assessment of drug interactions and toxicity.

## Methods

### Primary Hepatocyte Culture

Experiments in this study exclusively used 6-week-old, male Wistar rats weighing between 120 and 150 g. Animals were kept at a 12 h light/dark cycle with access to water and standard chow diet *ad libitum*. Animal handling and the experimental setup were in accordance with the University of Tokyo guidelines regarding animal experiments (approval number 2706).

Primary rat hepatocytes were isolated by standard two-step collagenase perfusion and subsequently purified by a series of washes with serum-free EMEM medium (Gibco, USA), followed by a Percoll (GE Healthcare, Sweden) gradient purification as previously described (Xiao et al., 2014). Immediately after the isolation, cells were plated at a density of 1.5–2.0 e5 cells/cm^2^ on Type 1-P porcine collagen-coated (Nitta Gelatin, Japan) 24 well tissue culture plates with oxygen-permeable PDMS bottom membranes (Vecell, Japan), which were surface-treated with silane-based crosslinking agents (Xiao et al., 2014) in order to improve cellular attachment. Inoculated Hepatocyte Plating Medium (Table 1a) was carefully aspirated after an initial incubation period of 3 h and replaced with Hepatocyte Maintenance Medium (Table 1b). Daily culture medium changes with maintenance medium were then further supplemented with 150 μg/ml growth factor reduced Matrigel (Corning, USA) in order to obtain a sandwich culture configuration.

**Table 1:** Medium composition for primary rat hepatocyte plating and maintenance.

**Component**	**Volume**	**Final concentration**	**Cat. No. (manufacturer)**
**a. Hepatocyte plating medium composition**			
Williams' E Medium	500 mL	–	#A1217601 (Gibco, USA)
Fetal Bovine Serum	25 mL	5%	#16000044 (Gibco, USA)
GlutaMAX (100X)	5 mL	2 mM	#35050061 (Gibco, USA)
HEPES pH 7.4 (1 M)	7.5 mL	15 mM	H3375 (Sigma, USA)
Penicillin-Streptomycin	5 mL	1X	#10378016 (Gibco, USA)
ITS-G (100X)	5 mL	1X	#41400045 (Gibco, USA)
Nicotinamide (5 M)	0.2 mL	0.2 mM	N0636 (Sigma, USA)
Dexamethasone (10 mM)	0.5 mL	1 μM	#041-18861 (Wako, Japan)
mEGF	0.25 mL	20 ng/mL	SRP3196 (Sigma, USA)
Ascorbic Acid (0.5 M)	0.5 mL	0.5 mM	#013-12061 (Wako, Japan)
**b. Hepatocyte maintenance medium composition**			
Williams' E Medium	500 mL	–	#A1217601 (Gibco, USA)
Sodium Pyruvate (100X)	5 mL	1 mM	#11360070 (Gibco, USA)
GlutaMAX (100X)	5 mL	2 mM	#35050061 (Gibco, USA)
HEPES pH 7.4 (1 M)	7.5 mL	15 mM	H3375 (Sigma, USA)
Penicillin-Streptomycin	2.5 mL	0.5X	#10378016 (Gibco, USA)
ITS+ (100X)	5 mL	1X	#354352 (Corning, USA)
Nicotinamide (5 M)	0.2 mL	0.2 mM	N0636 (Sigma, USA)
Dexamethasone (10 mM)	0.05 mL	0.1 μM	#041-18861 (Wako, Japan)
mEGF	0.25 mL	20 ng/mL	SRP3196 (Sigma, USA)
Ascorbic Acid (0.5 M)	0.5 mL	0.5 mM	#013-12061 (Wako, Japan)

After an adaption period of 24 h in a CO_2_ incubator with ambient oxygen levels to ensure comparable cellular attachment (Rotem et al., 1994), zonal phenotypes were induced by modulation of oxygen concentration and availability. In order to modulate oxygen availability, PDMS membranes of the culture vessels were either left as-is for directly oxygenated cultures [denominated as [+]] or blocked with an optical adhesion polyester film (Applied Biosystems, USA) to only allow oxygen supply by diffusion through the medium [denominated as [–]]. In detail, the polyester sealing was directly applied and adhered onto the PDMS membrane, mimicking the negligible gas diffusivity of conventional polystyrene tissue culture plates. Oxygen concentration was further varied between experimental groups by transferring cultures into multi-gas incubators with different oxygen levels (20, 10, 5, and 2.5%). To allow for the formation of stable phenotypes, hepatocytes were cultured for 5 days after induction, before being collected for analysis.

### Oxygen Consumption Measurement

In order to assess the Oxygen Consumption Rates (OCR) of individual phenotypes during the culture, oxygen partial pressures at the cellular level were registered after zonal induction, after 2 days, and after 5 days in culture by direct optical measurement with a REDFLASH-based fluorescent oxygen probe (PyroScience, USA). The probe was connected to a FireSting O_2_ logger (PyroScience, USA) and lowered onto the hepatocyte monolayer. Through red-light excitation of the sensor matrix, an oxygen-dependent phase shift during emission occurs near the probe. After reaching steady-state, the resulting emission of near-infrared light was then measured and recorded. With these values for pericellular partial pressures, oxygen consumption rates of each condition were calculated using the following equation based on Fick's law.

(1) $$\begin{array}{r} F=\left[{\left(\frac{DK}{h}\right)}_{Medium}+{\left(\frac{DK}{h}\right)}_{Material}\right]\left(C-C_{cell}\right)=n\cdot OCR \end{array}$$

Here, F represents the total oxygen flux entering the culture system. D is the diffusion coefficient of oxygen in the respective material, K is the material-dependent Henry constant, with h being the material thickness or diffusion length. C denominates the ambient oxygen concentration in the system, depending on the individual experimental condition. Accordingly, C_cell_ represents the pericellular oxygen concentration measured by the probe. Cellular oxygen consumption rates (OCR) are then calculated by the division of oxygen flux F with the cell density *n*. All parameter values are detailed in Table 2, based on the materials used in the respective condition. Diffusion in sealed PDMS [–] conditions is ideally assumed as 0, signified by diffusion coefficient D_Sealing_.

**Table 2:** Analytical parameters for computation of oxygen consumption rates.

**Material**	**PDMS**	**Sealing**	**Medium**
D	4.10E-05	0.00E+00	2.00E-05
K_H_	1.06E+04	1.06E+04	1.19E+03
h (cm)	0.1	0.1	0.25

### Drug Metabolism Assessment

For the assessment of phenotype-specific drug response, hepatocytes were subjected to 20 mM Acetaminophen (APAP) (Sigma Aldrich, USA) for 5 h. APAP was dissolved in maintenance medium and administered to the cells on the final day of culture. Duration and concentration of the treatment were chosen to mimic a therapeutic dose with expected minimal cellular necrosis according to previously published data (Jemnitz et al., 2008). After 5 h of exposure, the supernatant of both vehicle control, and treated cultures was collected for cytotoxicity assessment. Cellular damage caused by the drug treatment was then measured by LDH leakage quantification with an LDH-Cytotoxic Test kit (Wako-Fujifilm, Japan) according to the manufacturer's specifications. Here, LDH-dependent NADH production is measured through the reduction of a water-soluble tetrazolium salt to a formazan dye and subsequent colorimetric quantification at 540 nm.

### Quantification of Intracellular Nitric Oxide

Intracellular expression of reactive species between conditions was conducted with a single-laser MUSE Cell Analyzer (EMD Millipore, USA). Cells were detached from the culture vessels with 500 μl Accutase (Innovative Cell Technologies Inc, USA) cell detachment solution per well. Cultured hepatocytes were then stained with a Nitric Oxide kit (EMD Millipore, USA) according to the manufacturer's specifications. Measurement of Nitric Oxide (NO) expression was based on DAX–J2 Orange, which converts to a fluorescent product upon oxidation of NO. Quantification was conducted in combination with the cellular exclusion-based viability stain 7–Aminoactinomycin (7–AAD) in order to exclude non-viable cells.

### Extracellular Glucose and Lactate Quantification

In order to assess cellular carbon energy metabolism, culture supernatant was collected daily and analyzed using a BD-7D bioanalyzer (Oji Scientific, Japan). Quantification of extracellular Glucose and Lactate was carried out by electrochemical measurement of enzymatic hydrogen peroxide production with immobilized Glucose Oxidase and Lactate Oxidase electrodes, respectively.

### Enzyme-linked Immunosorbent Assay (ELISA) Measurement of Albumin and Insulin

Collected cell culture supernatant was further analyzed in terms of secreted albumin as a zonation marker by sandwich ELISA (Bethyl Laboratories, USA) according to the manufacturer's protocol. Microplates were incubated with a goat anti-rat Albumin capture antibody at 4°C overnight, washed with 0.1% Tween-20 in PBS, and then blocked with blocking buffer containing 1% bovine gelatin for 2 h at room temperature. Samples were then diluted appropriately in blocking buffer, added to the previously coated microplates, and incubated for 2 h at room temperature. After another washing step, plates were incubated with an HRP-conjugated sheep anti-rat albumin detection antibody for 2 h at room temperature. Following a final washing step, TMB (3,3′,5,5′-Tetramethylbenzidine) substrate solution was added and incubated for <10 min in the dark. Color development was then stopped by adding 1 M sulfuric acid and plates were immediately measured with a microplate reader (Bio-Rad. USA) at 450 and 570 nm.

Similarly, assessment of daily insulin uptake supplied from the culture medium was achieved with an anti-human Insulin sandwich ELISA (R&D Systems, USA) according to manufacturer's recommendations, including required assay buffers (R&D Systems, USA).

### Quantification of Urea Release

Secreted urea was quantified colorimetrically through enzymatic reaction with a commercial Urea Assay kit (Abcam, UK), following the manufacturer's instructions. The culture supernatant was diluted appropriately and incubated with the enzyme reaction mix at 37°C. After color development for 1 h, optical density was measured at 570 nm for sample quantification.

### Metabolomic Analysis

Intracellular metabolite concentrations were quantified using CE/MS separation and detection. After 5 days of differential oxygenation, both untreated and drug-treated groups were briefly washed twice with 500 μl 5% (w/v) aqueous D-Mannitol solution. Then, cells were rapidly lysed with 250 μl ice-cold methanol containing 25 μM of each internal standard [Methionine sulfone (Alfa Aesar, UK), 2-(N-morpholino)ethanesulfonic acid (MES, Dojindo, Japan), and D-Camphor-10-sulfonic acid (CSA, Wako Fujifilm, Japan)] for mass spectrometry analysis. After 10 min of incubation, methanol extracts were collected and stored at −80°C until further processing. Two hundred microliter of the extract was then mixed with 200 μl CHCl_3_ and 100 μl Milli-Q water, and centrifuged at 10,000g for 3 min at 4°C. Two hundred microliter of the aqueous layer was then transferred to a 5 kDa cutoff ultrafiltration tube (HMT Biomedical, Japan) and centrifuged at 9,100g for 2 h at 20°C. The resulting permeate was further concentrated for 2 h at 40°C. Prior to analysis, 12.5 μl Milli-Q water, containing 200 μM 3-Aminopyrrolidine (Sigma Aldrich, USA) and Trimesate (Wako Fujifilm, Japan) was added to each concentrate and then analyzed by CE-TOFMS (Agilent Technologies, USA) as previously described (Soga et al., 2009). Enrichment analysis of metabolite levels was performed with Metaboanalyst (www.metaboanalyst.ca) using the SMPBD metabolite set library and Fisher's exact test. Comparison of results was visualized with the R statistical language in terms of computed fold enrichment and Holm-Bonferroni adjusted *p*-values.

### Quantitative Real-Time PCR

Relative gene expression of hepatocyte phenotypes was assessed by quantitative real-time PCR. First, hepatocyte sandwich cultures were lysed in an appropriate amount of Trizol reagent (Thermo Fisher Scientific, USA), followed by RNA extraction using a silica column (Zymo Research, USA). Obtained total RNA was then quantified by Biospec-nano microvolume measurement (Shimadzu Scientific, Japan) and converted to complementary DNA with a PrimeScript reverse transcriptase kit (Takara, Japan). Individual primer sequences (Table 3) were designed with Primer-BLAST and purchased from Eurofins Genomics. Real-time PCR was performed with KOD SYBR Green chemistry (Takara, Japan) in a StepOnePlus (Applied Biosystems, USA) system, using β-Actin as an internal control. For detailed screening of cellular drug metabolism, RT^2^ qPCR Rat Drug Metabolism Panels, targeting 84 different related genes, were used with RT^2^ SYBR Green chemistry (Qiagen, Germany) according to manufacturer's recommendations. Similar to the analysis of individual targets, β-Actin was used as the reference gene.

**Table 3:** qRT-PCR primer sequences.

**Target**	**Forward sequence**	**Reverse sequence**
r_ALAT	CTGACTACTGGAGCGAGCGA	CTCGTCCACGTTCAAAGCCC
r_G6PC	CAACAGCTCCGTGCCTCTGA	CAGCCCAGTATCCCAACCACA
r_PCK1 (Cadoudal et al., 2007)	TGTTGGCTGGCTCTCACTG	ACTTTTGGGGATGGGCAC
r_PGM1	GGAAGCGAGTGAAGGTGTTCCA	TCCAATAACCAGGCGGCCAA
r_PDH	AGGAAGATGCTTGCCGCTGTG	TTACGGGAAGCTACCAGCACTC
r_PC	GTTCCCTGTCAGTGGAGGCA	TGGGCTTGTACTCCAGACGC
r_ARG1 (Walters and Wallace, 2010)	GCGTCATTTGGGTGGATGCT	GTCCACATCTCGCAAGCCGA
r_CPS1 (Walters and Wallace, 2010)	TGAGACAGGCCAAAGAGATTGGGT	TGCTCCTGGCCATTGTAGGTAACA
r_OTC	TCCGCGGTCATTAGTGTTCC	GCAGGCATCAGAACTTTGGC
r_SULT1A1	AAGCTAGAGAAGTGTGGCCG	ACCACGACCCATAGGACACT
r_UGT1A1	TGGGTCACTTGCCACTGAAAT	AGGCGTTGACATAGGCTTCA
r_ACTINB	AGAGAAGCTGTGCTAT	GTACTCCTGCTTGCTGATCC

### Immunocytochemistry

After 5 days of culture post-induction, hepatocytes were fixed with 4% Paraformaldehyde in PBS for 10 min at room temperature. Cells were then permeabilized with 0.5% Triton X-100 dissolved in PBS for 5 min at room temperature, followed by three washes with 1X PBS for 5 min each and incubation in blocking solution containing 1X PBS, 5% normal donkey serum, 0.01% Tween-20, and 0.3 M Glycine for 1 h. Samples were then incubated overnight at 4°C with primary antibodies diluted in PBS, 1% BSA, and 0.01% Tween-20. The following primary antibodies were used: rabbit anti-*glutamine synthetase* (1:200, ab49873, Abcam, UK); sheep anti-*SLC2A1* (1:200, ab54263, Abcam, UK; sheep anti *CDH2* (1:400, AF6426, R&D Systems, USA); rabbit anti-*active* β*-catenin* (1:800, D13A1, Cell Signaling, USA). After repeated washing steps, diluted (1:500) secondary antibodies conjugated with Alexa fluorochromes were incubated for 3 h at room temperature in the dark. For this, donkey anti-rabbit AlexaFluor 568 (ab175692, Abcam, UK), and donkey anti-sheep AlexaFluor 647 (ab150179, Abcam, UK) were utilized. Samples were then incubated with DAPI (Donjindo, Japan) diluted 1:1,000 in PBS for 10 min at room temperature, washed with PBS 3 times for 5 min each, and mounted to cover glasses with Prolong Diamond (Thermo Fisher, USA).

Immunofluorescent staining was imaged with an FV3000 confocal microscope, using a UPLS-APO 100XO oil-immersion objective lens (Olympus, Japan). Obtained Z-stack images were deconvolved with the cellSens Dimension software suite (Olympus, Japan) using the Wiener deconvolution function. Comparisons show representative images from each observed condition.

### Statistical Analysis

Results are represented as mean ± S.D. of *n* = 3 independent experiments. Statistical comparison was performed by one-way ANOVA followed by Tukey's HSD *post-hoc* test for individual comparison between conditions. Pairwise comparisons were conducted via Student's *t*-Test.

## Results

### Modulation of Oxygen Consumption Independent of Ambient Oxygen Tension

**Figure 1:**
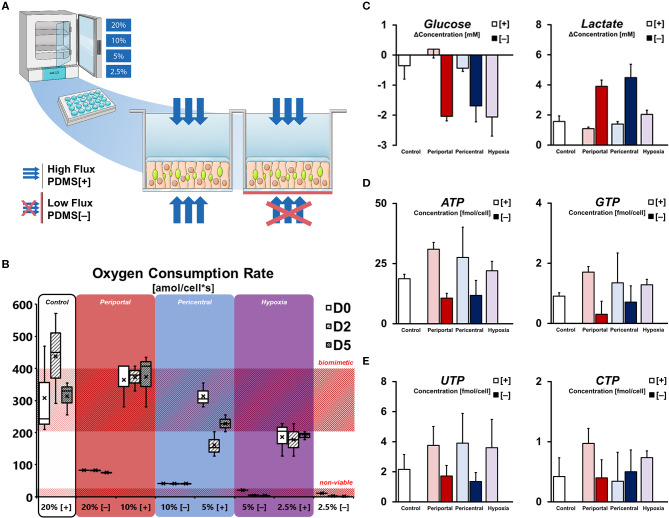
Microenvironmental effect on cellular energy balance. **(A)** Experimental setup for differential oxygenation of primary rat hepatocytes. Gas-permeable culture plates are used for high [+] oxygen flux. Selective blocking with polyester seals [–] limits gas diffusion to the medium side only. Further variation of oxygen tension is achieved by multi-gas incubators with ambient pO_2_ set to 20, 10, 5, and 2.5%. **(B)** Cellular oxygen consumption rates (OCR) calculated from pericellular oxygen measurement. High flux conditions allow OCR in the range of previously reported *in vivo* values (Place et al., 2017) while blocked cultures are only viable in 20 and 10%. **(C)** Purine triphosphate balance and **(D)** pyrimidine triphosphate balance in conditions subjected to differential oxygenation. **(E)** Supernatant glucose and lactate concentration change per day on D5.

In order to modulate hepatocellular oxygenation, primary hepatocyte cultures were exposed to different ambient oxygen levels in regulated multi-gas incubators. Gas-permeable culture vessels allow for precise regulation of cellular oxygen levels, however, they are generally not used in routine applications involving hepatocyte cultures. By blocking the bottom membrane of selected cultures, oxygenation similar to conventional tissue culture plates was achieved, limiting the oxygen supply to diffusion through the aqueous medium—while ensuring equal cellular setups between conditions in terms of cell density and physical quality of materials. These setups were subsequently cultured in incubators with ambient oxygen levels ranging between 20 and 2.5% (Figures 1A, S1a) with the intent of generating defined, oxygen-dependent phenotypes mimicking zonation-like metabolic patterns. Due to the difference in oxygen levels and supply rate, we expected cultures to assume phenotypes based on general oxygen availability. We, therefore, defined individual culture conditions as follows: “Control” cultures in 20% oxygen with gas-permeable membranes (PDMS [+]), were compared to cultures in reduced ambient oxygen levels—with 10% mimicking periportal, 5% pericentral, and 2.5% hypoxic oxygen concentrations. Conversely, cultures with blocked bottom membranes (PDMS [–]) were defined as periportal phenotypes in high oxygen levels (20%) and pericentral in the lowest viable oxygen concentration of this setup (10%).

Direct measurement of pericellular oxygen levels allowed for the calculation of condition-specific oxygen consumption rates. Whereas, PDMS [+] cultures in high ambient oxygen (i.e., 20% [+] and 10% [+]) exhibited OCRs of around 400 amol/cells, the measurement of cells in low ambient oxygen showed decreased values at around 200 amol/cells (Figure 1B). Interestingly, cells in 5% [+] appeared to change their oxygen consumption over the course of the culture duration as evident by the unstable OCR values over time. In contrast, PDMS [–] conditions globally exhibited stable, but lower oxygen consumption rates—generally consuming all available oxygen in the culture medium and resulting in depleted pericellular oxygen levels (Figure S1b). OCR of viable conditions was measured at 88 and 44 amol/cells for 20% [–] and 10% [–], respectively, while cultures in 5% [–] and 2.5% [–] were considered non-viable (Figure 1B). Comparison of both setups further allowed to directly assess the limitation of cellular respiration in conventional culture conditions. In the viable low flux conditions 20% [–] and 10% [–], cells were limited to 25 and 12% of their uninhibited respiration rates in the related PDMS [+] conditions, respectively (Figure S1c).

Based on cellular respiration, we considered the basic metabolic phenotypes of the individual conditions in terms of their energy metabolism (Figure 1C). Measuring the changes in glucose and lactate concentration in the culture supernatant revealed distinct gradual patterns in high flux conditions. Whereas, hepatocytes in 10% [+] were able to achieve a net production of glucose, cultures in lower ambient oxygen concentrations were found to gradually increase their glucose consumption. Similarly, a gradual increase in lactate in the culture medium was observable with lower oxygen concentrations in those groups. In contrast, hepatocytes in PDMS [–] showed high levels of glucose consumption and lactate production, with no significant distinction between groups (*p* = 0.423 and *p* = 0.444, respectively). Based on these metabolic conditions, the observation of energy-dependent, intracellular nucleotide levels is consistent. Purine triphosphates (ATP, GTP) levels were generally higher in PDMS [+] cultures compared to low flux conditions, while further exhibiting a gradual decrease of mean metabolite concentration between 10% [+] and 2.5% [+] (Figure 1D). Notably, ATP concentrations in the control groups were significantly lower (*p* = 0.007) than the induced periportal phenotype in 10% [+], indicative of possible metabolic self-regulation in hyperoxic conditions. In terms of pyrimidine triphosphate balance, both UTP and CTP exhibited less distinct differences. Even though UTP content was still globally higher in PDMS [+] conditions, levels of CTP were not substantially different (Figure 1E).

### Zonation-Like Profiles of Glucose Metabolism Require Sufficient Oxygen Supply Rates

**Figure 2:**
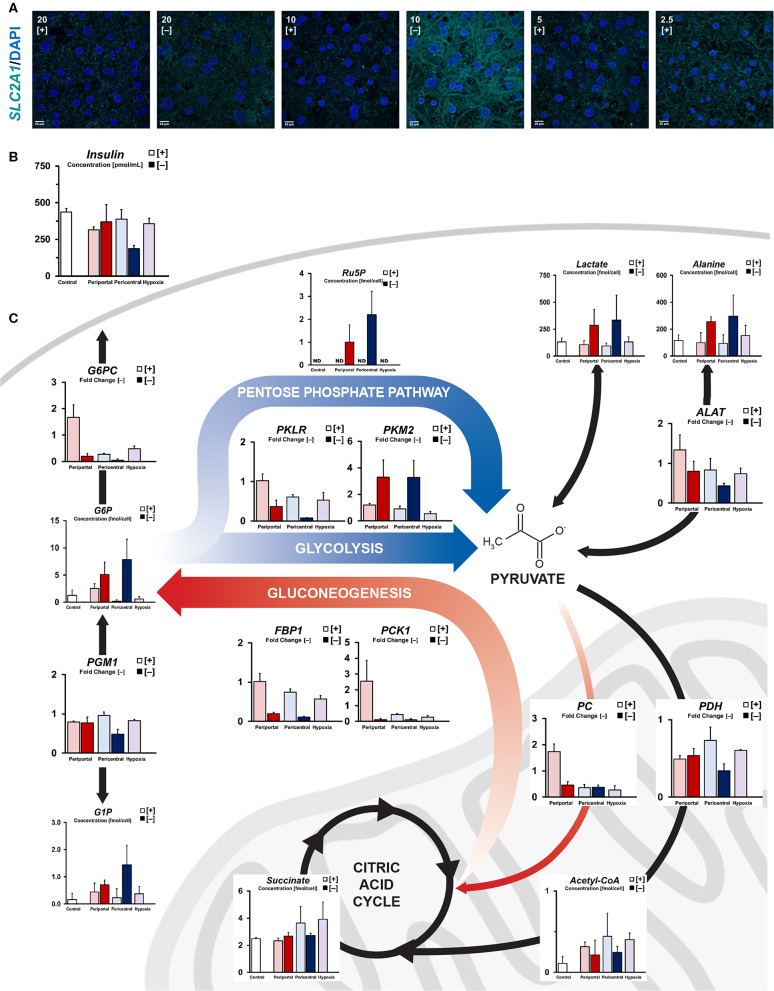
Quantification of intracellular carbon energy metabolism. **(A)** Immunofluorescent staining of *SLC2A1* expression in different oxygen conditions. Translocation to the plasma membrane indicates active glucose transporter function (scale bar = 10 μm). **(B)** Residual supernatant insulin concentration after 24 h of culture at day 5. **(C)** Simplified carbon energy metabolism showing phenotype-specific metabolic patterning. High flux periportal-like cultures exhibit increased gluconeogenetic capabilities, whereas pericentral and hypoxic cultures accumulate citric acid cycle intermediates. Low flux cultures ambiguously exhibit cytosolic metabolism with *PKM2*-dependent pentose phosphate pathway activation.

Considering individual energy levels, we assessed cellular carbon metabolism in more detail. Based on glucose consumption levels, we observed increased levels of cytosolic energy metabolism in PDMS [–] cultures. Consistently, high-affinity glucose influx transporter *SLC2A1* was noticeably translocated to the plasma membrane in 10% [–] conditions (Figure 2A). Furthermore, we found significantly increased (*p* = 0.018) cellular uptake of insulin (Figure 2B), whereas other conditions retained comparable concentrations in the culture medium. Accordingly, increased glucose influx in PDMS [–] conditions, as well as subsequent intracellular metabolite levels of cytosolic carbon compounds (Figure 2C) are consistent with observed supernatant concentrations of glucose and lactate (Figure 1C). As such, we found key metabolites to be upregulated in both catabolic and proliferative pathways. Regarding glycolytic carbon utilization, accumulation of glucose-6-phosphate (G6P), intracellular lactate and L-alanine were observed in PDMS [–] conditions, with a higher mean concentration in lower ambient oxygen levels. In terms of cellular proliferative pathways, analogous accumulation of glucose-1-phosphate (G1P) and ribulose-5-phosphate (Ru5P) suggest the involvement of glycogenesis and the pentose phosphate pathway, respectively. Even though G1P concentrations, as well as elevated insulin signaling, suggest increased rates of glycogenesis, limited availability of UTP (Figure 1E) acts as a bottleneck in this pathway, resulting in similar levels of glycogen precursor UDP-glucose (Figure S1e). In contrast to energy-storing glycogenesis, the pentose phosphate pathway supplements cellular homeostasis through the generation of NADPH and pyruvate. Additionally, amino acid metabolism appears to supplement cellular glucose consumption based on elevated intracellular L-alanine levels (Figure 2C). Notably, similar patterns were not observed in terms of mRNA expression (Figure 2C). *PGM1* (*p* = 0.016) and *PKLR* (*p* = 0.002) gene expression were significantly downregulated in low flux conditions, thus limiting G6P and G1P interconversion, as well as glycolytic pyruvate generation. Instead, a metabolic shift in the final step of glycolysis was observed via significant upregulation of the *PKM2* isoform of pyruvate kinase (*p* = 0.022).

In comparison, PDMS [+] groups exhibited generally increased levels of mitochondrial metabolism and lower levels of cytosolic intermediates (Figure 2C, Figure S1e) based on their elevated oxygen consumption (Figure 1B). With cytosolic metabolites mostly showing little difference between high flux conditions, only glucose-6-phosphate levels were considerably elevated in high oxygen cultures compared to other lower ambient oxygen tension conditions. Similarly, key gluconeogenetic genes showed zone-specific upregulation in 10% [+] cultures (*PC, PCK1, FBP1, G6PC*), consistent with observed medium glucose content (Figure 1C). While periportal conditions exhibited gluconeogenetic abilities, cultures at lower ambient oxygen tension instead exhibited mitochondrial accumulation of carbon structures such as succinate, fumarate, and malate (Figure 2C, Figure S1e). Supplementation of these metabolites via *PDH*-dependent utilization of pyruvate was consistent with previously observed high respiratory rates across PDMS [+] cultures according to similar expression levels between conditions. Instead, *PDH* gene expression was found to be downregulated between pericentral conditions (*p* = 0.043) in 10% [–], in line with the carbon distribution in those conditions.

In brief, high flux conditions exhibited gluconeogenetic gene and metabolite profiles in periportal oxygen levels, whereas reduced oxygen tension led to an accumulation of citric acid cycle intermediates. Conversely, low flux conditions showed an elevated concentration of cytosolic metabolites, as well as a metabolic shift in glycolysis via *PKM2* induction.

### Oxygen Limitation Alters Nitrogen Metabolism in an Energy-Dependent Manner

**Figure 3:**
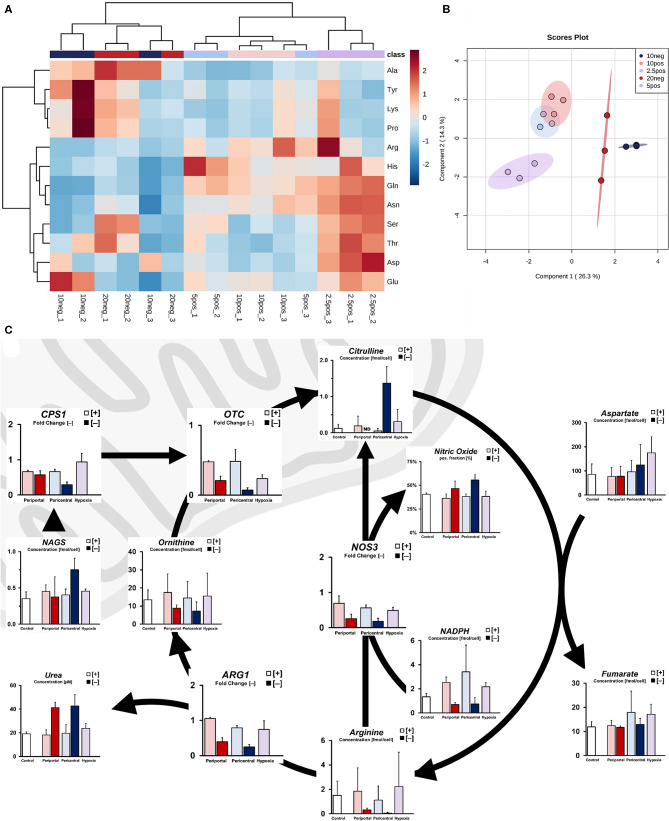
Ischemic effect on nitrogen metabolism due to limited oxygen availability. **(A)** Intracellular amino acid profile of individual cultures as fold accumulation compared to control conditions **(B)** sPLS-DA principal component analysis based on cellular amino acid profiles. **(C)** Simplified pathway of cellular ammonia detoxification showing transcript level-independent urea cycle activity in low flux conditions, resulting in increased nitrogen efflux via urea and nitric oxide.

With increased alanine levels indicating amino acid-dependent supplementation of pyruvate in low flux conditions, we further investigated the nitrogen metabolism of induced phenotypes. While cellular carbon energy metabolism was mostly distinguishable in periportal high flux conditions compared to lower oxygen tensions, nitrogen metabolism instead showed differences between normoxic and hypoxic conditions. Amino acid profiles in normoxic PDMS [+] conditions (10 and 5%) were largely similar, clustering in a non-specific manner without distinct accumulation patterns (Figure 3A). In contrast, we observed a distinguishable hypoxic phenotype exhibiting elevated levels of acidic amino acids (Asp, Glu) and their derivatives (Asn, Gln). Additionally, other glucogenic amino acids (Ser, Thr, His, Arg) were similarly accumulated. Metabolite levels observed in low flux conditions, on the other hand, contrasted the previously described hypoxic profile. These amino acids were generally depleted with the exception of Glu in 10% [–], as well as Ser and Thr in 20% [–] conditions. Instead, we observed distinct accumulation of Ala, Tyr, Lys, and Pro (Figure 3A). Despite these amino acids being accumulated in similar ways in both low flux conditions, phenotypes could not be distinguished via hierarchical clustering—instead indicating a combined ischemic phenotype in comparison to high flux cultures (Figure 3B).

Based on these amino acid profiles, we next considered differences in ammonia detoxification processes of the urea cycle. Although we found no substantial difference between mRNA levels of genes involved in the urea cycle under PDMS [+] conditions, transcripts of these genes were downregulated in PDMS [–] cultures (Figure 3C). Increased metabolite levels of N-acetylglutamate in low flux conditions, however, indicate increased activity of ammonia detoxification via *CPS1* activation (Figure 3C). Consistently, we observed higher urea secretion in these samples compared to PDMS [+] groups. Moreover, intracellular levels of citrulline were significantly elevated (*p* = 0.007) in 10% [–] cultures, caused either by upregulated ammonia entry into the urea cycle or production via nitric oxide synthase from arginine. Indeed, flow cytometric analysis revealed significantly increased expression of nitric oxide (Figure 3C) in low flux cultures (*p* = 0.023), regardless of oxygen-dependent attenuation of *NOS3* gene expression. We further observed reduced levels of the required cofactor for this reaction, NADPH (Figure 3C). Hence, this indicates increased urea cycle activity independent of transcript levels and subsequently elevated ammonia detoxification rates in low flux conditions.

### Morphogenic Signaling and Assumption of Functional Zonation

**Figure 4:**
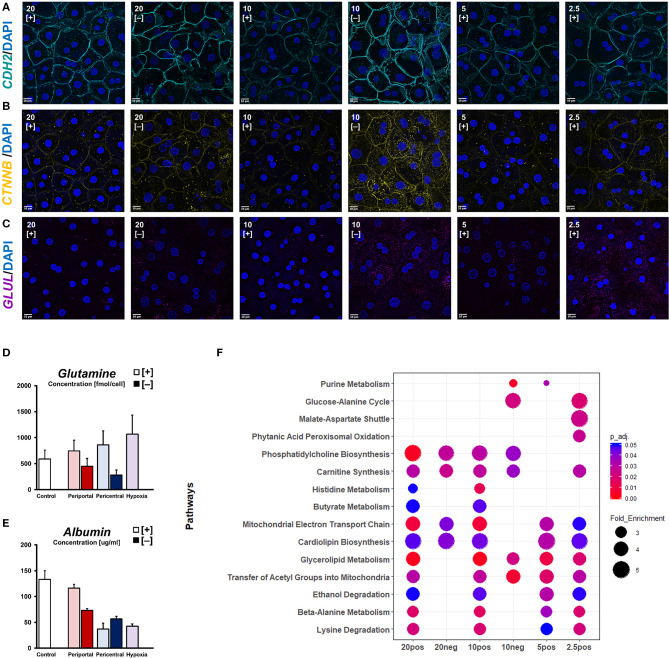
Oxygen-dependent signaling induces zonation-like phenotypes. Immunofluorescent imaging of **(A)** *CDH2*, **(B)** *active* β*-catenin (CTNNB)*, and **(C)** *glutamine synthetase* (*GLUL*) observed in different conditions (scale bar = 10 μm). **(D)** Intracellular glutamine levels and **(E)** secreted albumin levels after 5 days of differential oxygenation. **(F)** Comparative metabolomic overrepresentation analysis of untreated cultures after 5 days of culture.

With basic cellular functions clearly modulated in response to differential oxygenation, we sought to characterize individual phenotypes in terms of cellular signaling pathways related to pericellular oxygen conditions. Considering β-catenin as one of the most important morphogens regarding metabolic zonation, we investigated its cellular availability for signaling pathways. Active, non-phosphorylated β-catenin was observed near the plasma membrane in all conditions, co-localizing with cellular adhesion molecules, like N-Cadherin (*CDH2*) (Figure 4A). Additionally, we observed cytosolic protein expression needed for nuclear translocation of active β-catenin signaling in hypoxic high flux conditions, as well as in 20% [–] and 10% [–] conditions (Figure 4B). Accordingly, glutamine synthetase—a β-catenin transcriptional target—was similarly expressed in 10% [–] and 2.5% [+] conditions (Figure 4C). Based on the expression of signaling proteins in low oxygen conditions, assessment of metabolite levels for the zonation landmark genes albumin and glutamine reveal oxygen-dependent gradients (Figures 4D,E). Intracellular glutamine content is consistent with the observed expression of glutamine synthetase, increasing gradually in lower ambient oxygen in high flux conditions (Figure 4D). In low flux conditions, however, a decrease in ambient oxygen levels also decreases cellular glutamine levels, contrasting cellular glutamine synthetase expression (Figures 4C,D). Conversely, we observed distinguishable albumin secretion between high expression in 20 and 10% pO_2_, and low expression in 5 and 2.5% pO_2_ when considering PDMS [+] cultures (Figure 4E). A similar profile was also observed in PDMS [–] conditions, discriminating between high periportal, and low pericentral expression. In order to estimate the time required to induce phenotypes, we further considered the time-dependent changes in both functional and metabolic biomarkers. The reduction of ambient oxygen tension in high flux conditions led to a time-dependent decrease in supernatant albumin levels. Daily secretion generally stabilized on the previously described profiles after 3 days of differential oxygenation (Figure S2a), while control hepatocytes retained a stable phenotype for the whole culture duration. In contrast, reducing oxygen supply rates immediately decreased albumin secretion rates in low flux conditions. Compared to the stable functionality of periportal cultures over time, pericentral conditions slowly increased their secretion. Likewise, energy metabolism appears to gradually adapt post-induction (Figures S2b,c). Both glucose consumption and subsequent lactate production increases over time in PDMS [–] conditions and reaches a steady-state after around 4 days. Similarly, PDMS [+] conditions conform to previously discussed glucose consumption profiles toward the last day of culture (Figure S2b). In contrast, lactate production in these conditions remains statistically constant in all groups (Figure S2c). Based on intracellular metabolites, we further estimated cellular functional differences via overrepresentation analysis (Figure 4F). While phosphatidylcholine biosynthesis was found to be overrepresented based on high ambient oxygen tension, pathways regarding beta-alanine metabolism, lysine, and ethanol degradation appeared dependent on high cellular OCR. Furthermore, histidine and butyrate metabolism were likely influenced by a combination of those factors. In contrast, functions regarding amino acid metabolism were enriched in lower respiratory cultures. Notably, low flux conditions did not exhibit enriched metabolite levels of mitochondrial pathways compared to PDMS [+] groups.

### Drug Metabolic Functionality of Zonation-Like Phenotypes

**Figure 5:**
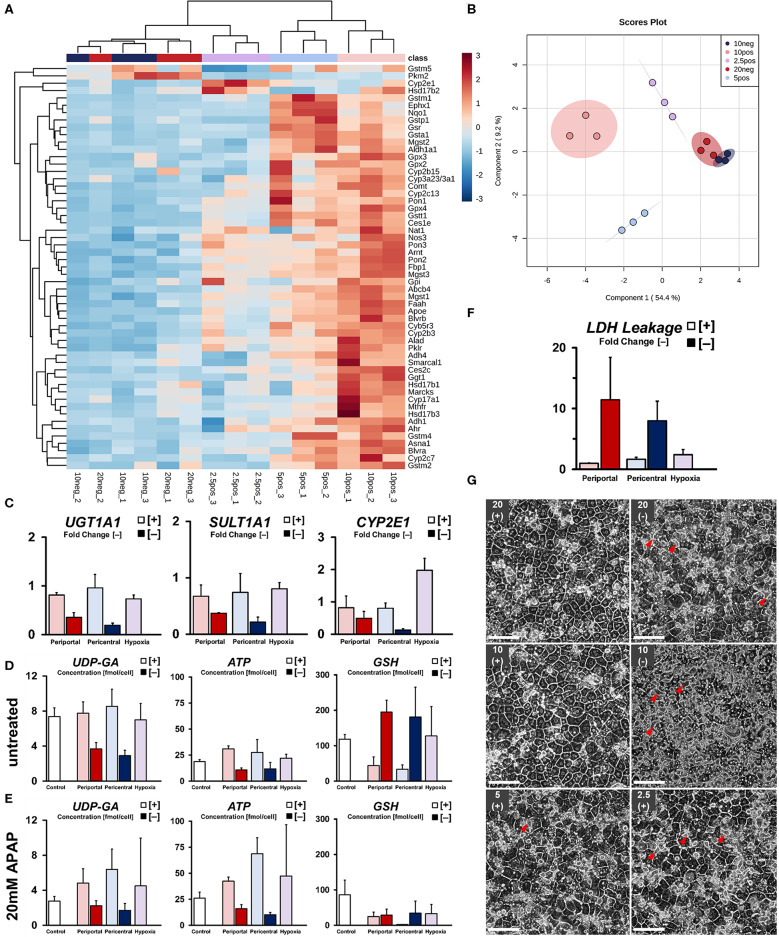
Drug metabolic capabilities of induced phenotypes. **(A)** Differential expression of drug metabolism-related genes in induced phenotypes as fold change versus control groups. **(B)** sPLS-DA principal component analysis of drug metabolism-related phenotypes. **(C)** Summary of relevant gene expression for APAP drug metabolism and transport. Quantification of related co-factors shown in terms of intracellular concentration of UDP-glucuronate for UGT1A1-mediated glucuronidation. ATP-availability required for ATP-dependent drug efflux and as a precursor for SULT1A1-related drug detoxification. Intracellular availability of reduced glutathione required for detoxification of monooxygenase-derived drug intermediates in **(D)** untreated and **(E)** 20 mM APAP treated groups. **(F)** Quantification of cytotoxicity after drug exposure compared to control groups. **(G)** Cellular morphology of hepatocyte sandwich cultures after exposure to 20 mM Acetaminophen (APAP) for 5 h on day 5 (scale bar = 100 μm). Arrowheads show pathological expansion of bile canaliculi.

Next, we investigated the capability of cellular phenotypes to metabolize xenobiotics, as well as the expression of related genes. Globally, cells cultured under high flux conditions within normoxic ranges exhibited higher relative gene expression (Figure 5A) of drug metabolism-related enzymes (*CYB5R3, CYP3A2, CES1E*). Upregulated gene expression in periportal-like conditions, on the other hand, was observed regarding glucocorticoid and steroid metabolism (*HSD17B1, HSD17B3, CYP17A1*), as well as cellular energy metabolism (*FBP1, PKLR*). A gradual decrease of transcript abundance of these periportal genes was found to extend toward hypoxic conditions in an oxygen tension-dependent manner. Within high flux groups, distinct clusters of genes were upregulated in lower oxygen conditions, therefore excluding strict hypoxia-related downregulation of transcript levels. In pericentral conditions, those genes mostly related to phase II drug metabolism regarding glutathione (*GSR, GSTM1, GSTA1*) and NADPH (*NQO1*) consistent with lower homeostatic antioxidant requirements in lowered oxygen tension. These genes, however, were sharply downregulated in hypoxic cultures. Instead, significant upregulation of the related phase I monooxygenase *CYP2E1* (*p* = 0.0011) was observed in this condition. Whereas, high flux conditions could clearly be discriminated between one and another—clustering in normoxic and hypoxic groups as previously described—cultures exposed to lower oxygen supply rates exhibited generally reduced gene expression without the assumption of distinguishable phenotypes. Instead, we observed one superseding ischemic phenotype, most closely related to hypoxic cultures. Principal component analysis confirmed these findings (Figure 5B), showing overlapping confidence intervals of low flux groups. Globally, individual phenotypes were characterized in relation to their observed OCR along principal component PC1. Within normoxic oxygen levels, high flux periportal and pericentral conditions could further be separated according to PC2.

In order to assess individual drug metabolic capabilities, we next subjected all conditions to 20 mM Acetaminophen (APAP) for 5 h. Comparison of relevant drug metabolic gene expression (Figure 5C), as well as metabolite levels of involved co-factors and their precursors (Figures 5D,E), shows general attenuation of the main, non-toxic drug metabolic pathways via glucuronidation and sulfation in low flux conditions. Rather, availability of glutathione and subsequent reduction in metabolite concentration after drug exposure suggests drug metabolism mainly via CYP450 oxidation (Figure 5E). Cultures in high flux conditions, on the other hand, exhibited a generally higher abundance of co-factors and pre-cursors required for homeostatic pathways, as well as higher gene expression levels regarding glucuronidation and sulfation of APAP. Even though we found significant upregulation of the relevant CYP450 monooxygenase *CYP2E1* in hypoxic cultures (Figure 5C), subsequent phase II conjugation with glutathione was substantially downregulated in comparison to higher oxygen conditions. Consistently, residual intracellular glutathione levels after drug exposure were found substantially increased compared to the observed depletion (Figure 5E) in pericentral cultures. Quantification of drug-induced cellular damage revealed a gradual increase in cytotoxicity with decreasing oxygen tension in high flux conditions. In contrast, low flux cultures exhibited strongly increased LDH leakage (Figure 5F) regardless of individual condition. Although drug-induced damage was not further statistically distinguishable, observed changes in cellular morphology were showing more nuanced details in this regard. High flux cultures in high ambient oxygen levels exhibited little change in morphology after drug exposure (Figure 5G). However, in comparison, hepatocytes subjected to hypoxic conditions exhibited slight bile canalicular expansion. Notably, cultures in low flux setups were showing similar, but increased structural changes. In the 10% [–] condition, this effect was observed homogeneously throughout the culture, similar to an induced loss of cellular adhesion. Consistent with this increasing cellular injury, cultures in low oxygen conditions were enriched in metabolites of the antioxidant-generating pentose phosphate pathway (Figure S1d). Low flux conditions further exhibited increased levels of energy-related metabolites, such as acetyl-CoA, citrate, and fumarate—indicating increased energy requirements in these cells (Figure S1e).

Similarly, the intracellular concentration of most amino acids was elevated (Figure S1e), even though urea cycle intermediates, such as ornithine and arginine were reduced compared to high flux conditions. Instead, cultures subjected to high oxygen supply rates exhibited increased levels of proliferative and cytoprotective metabolites. Energy-dependent nucleotides ATP, CTP, and GTP, as well as antioxidant NADPH, and the glutathione precursors S-Adenosylmethionine were observed at elevated levels (Figure S1e), indicative of cellular health in these conditions.

## Discussion

In this work, we investigated the influence of differential oxygenation in primary hepatocyte cultures both in terms of ambient oxygen concentration and supply rates in order to induce distinct phenotypes mimicking metabolic zonation. By utilizing gas-permeable culture vessels, we were able to meet the oxygen demand of these highly metabolically active cells, while directly modulating their pericellular oxygen tension. Comparing these tissue cultures to conventional cell culture setups has shown the importance of adequate oxygen supply rates regarding cellular metabolism and phenotypic gene expressions, highlighting the contrast between reduced functionality of low flux, and zonation-like phenotypes in high flux cultures.

Even after prolonged culture, cells experiencing high oxygen supply rates were able to maintain their oxygen consumption rates within the range of freshly isolated hepatocytes (Place et al., 2017), indicating metabolic stability in biomimetic environment. Despite pericellular oxygen not being a limiting factor, cultures modulated their OCR according to the ambient oxygen levels, suggesting an inherent effect of oxygen tension on cellular metabolism regardless of maximal possible metabolic capability. Indeed, hypoxia signaling is known to regulate cellular carbon energy metabolism by repressing gluconeogenetic gene profiles in hepatocytes (Ramakrishnan and Shah, 2017). Consistent with this effect, periportal hepatocytes *in vivo* exhibit higher capabilities to produce glucose with a gradual decrease toward pericentral areas (Kietzmann, 2017) similar to our induced phenotypes (Jungermann and Kietzmann, 2000). Accumulation of key glycolytic metabolites like succinate and extracellular lactate in reduced oxygen levels are implicated in further stabilization and activation of hypoxia signaling molecules (Haas et al., 2016). While cellular carbon energy metabolism showed clear periportal bias according to our defined conditions, differential nitrogen metabolism was instead mostly observed between normoxic and hypoxic conditions. In general, hepatic nitrogen metabolism is distinguishable between periportal ammonia detoxification and pericentral nitrogen recycling *in vivo* (Kietzmann, 2017). Modulation of oxygen tension in high flux conditions, however, did not yield distinct phenotypes in terms of urea cycle gene expressions. Genes involved in this pathway have been shown to be regulated via glucagon signaling (Gebhardt and Mecke, 1979), one of the prevalent endocrine signals known to induce periportal hepatocyte phenotypes (Cheng et al., 2018). Previous research (Krones et al., 1998) has further shown the oxygen-dependency of glucagon receptor induction in primary rat hepatocyte cultures, resulting in increased signaling effects in periportal culture conditions. However, due to the culture medium lacking glucagon, phenotype-specific signaling based on pericellular oxygen tension could not be induced, showing the requirement of systemic consideration for *in vitro* zonation. In contrast, metabolomic analysis revealed distinct amino acid accumulation in hypoxic conditions. Since amino acids like aspartate and asparagine are crucial for hypoxic cell metabolism (Garcia-Bermudez et al., 2018), their influx is specifically elevated in pericentral hepatocytes (Berger and Hediger, 2006). Additionally, nitrogen scavenging via glutamine production is an important, zone-specific ability of pericentral hepatocytes *in vivo*. While our defined and physiological phenotypes did not overlap completely in this case, an oxygen-dependent change in metabolic functions can, nevertheless, be observed.

Cultures in low flux conditions, on the other hand, exhibited OCR values limited by the diffusion rate through the medium due to pericellular oxygen depletion. This leads to cellular metabolic changes to adapt to adverse conditions. In these ischemic conditions, the observed accumulation of extracellular lactate indicates a metabolic shift toward anaerobic pathways. Consistently, increased glucose consumption sustains the resulting cytosolic carbon metabolism. Despite similar total glucose consumption in low flux groups, *SLC2A1* membrane translocation and increased insulin uptake in highly ischemic conditions indicate a need for higher basal glucose influx in conventional culture systems. Even though *SLC2A1* is generally considered to be an insulin-independent glucose transporter (Ebeling et al., 1998), membrane translocation has previously been observed in an insulin-dependent manner during ischemia in cardiomyocytes (Egert et al., 1999), similar to the presented culture conditions. Notably, this increased utilization of glucose appears to not explicitly serve glycolysis. Instead, upregulation of the pyruvate kinase isoform *PKM2* is known to slow down glycolytic processes (Cairns et al., 2011) in order to enable carbon structures to enter other proliferative pathways like the pentose phosphate pathway. This metabolic reprogramming follows a pattern known to ameliorate ischemic injury. Ammonia-dependent NO generation due to amino acid catabolism induces insulin signaling (Guarino et al., 2003), causing a metabolic shift toward *PKM2* expression (Iqbal et al., 2013) and the subsequent reduction of glycolytic flux (Cairns et al., 2011) for redirection of metabolites to proliferative pathways. Energy generation is then further fueled through amino acid catabolism, causing a feedback loop to stabilize this condition. Additionally, NO directly inhibits *PKM2* activity at the protein level (Zhou et al., 2019), further ensuring substrate availability for the NADPH generation. While enabling sufficient cellular redox balance in ischemic cells, this pathway is also commonly found in hepatocellular carcinoma (HCC) (Yang and Lu, 2013; Wong et al., 2014), implicating these metabolic changes to early-stage carcinogenesis. Indeed, prolonged ischemia has been linked to the recurrence of HCC (Grat et al., 2018), reducing the value of low flux conditions for homeostatic modeling of zonation-like hepatocytes.

Zonal phenotypes not only relate to individual cellular metabolism, but also to the collective tissue functionality induced through cellular signaling. In general, Wnt/β-catenin signaling—the “master regulator of zonation” (Burke and Tosh, 2006)—depends on the binding of extracellular Wnt ligands and the subsequent inactivation of the β-catenin phosphorylation complex (Yang et al., 2014). However, since expression of the “zonation keeper” *APC* (Benhamouche et al., 2006), a component of this degradation complex, is regulated by ambient oxygen tension (Newton et al., 2010) non-canonical signaling without Wnt ligands has been argued (Matsumoto et al., 2019) to influence pericentral zonation-like profiles *in vitro*. Even though many signaling pathways involved in metabolic zonation are inherently oxygen-dependent (Newton et al., 2010), results indicate that specific β-catenin activation in monocultures might only occur outside of the physiological range in the context of hepatic oxygen tension (Kietzmann, 2017). Instead, supraphysiological pericellular oxygen tension was required for cytosolic expression of active β-catenin and subsequent expression of glutamine synthetase (Burke and Tosh, 2006). Notably, oxygen-dependent β-catenin signaling was also induced in ischemic culture conditions at increased rates, proportional to observed pericellular oxygen tension. Consistently, this follows the previous argumentation regarding oncogenic metabolic phenotypes (Loeppen et al., 2002) in low flux conditions, as excessive activation of β-catenin is a key characteristic of carcinogenesis (Polakis, 1999). Thus, extracellular Wnt ligands seem to be an important factor to consider for the physiological induction of pericentral phenotypes in hepatocellular systems *in vitro*. Increasing appreciation regarding the involvement of non-parenchymal cells in hepatic zonation (Zeng et al., 2007; Halpern et al., 2018) has greatly improved the understanding of paracrine Wnt signaling due to zonal subpopulations of liver endothelial cells (Halpern et al., 2018). Proper translation of this phenomenon *in vitro* would thus require the inclusion of non-parenchymal cells, restoring complexity in simplified toxicological assays. This consideration is especially relevant in terms of hepatic drug metabolism, as many involved genes are direct downstream targets of β-catenin signaling (Gerbal-Chaloin et al., 2014).

Drug metabolic phenotypes largely depend on transcriptional regulation to induce gene patterns, however, metabolite availability for xenobiotic detoxification requires equal attention for physiological models. Compared with low expression of key drug metabolic genes in low flux conditions, which mimicked ischemic attenuation *in vivo* (Angus et al., 1990), normoxic high flux conditions enabled globally increased expression of drug metabolism-related genes. These normoxic cultures largely assumed zonation-like phenotypes according to our previously defined criteria. Notably, we found non-zonated genes (Halpern et al., 2017) to be only stably expressed in this oxygen range. This narrow physiological range must further be highlighted by the β-catenin dependent induction of *CYP2E1* in hypoxic high flux conditions. Drug metabolism mediated by this enzyme requires further phase II conjugation for cellular efflux reactions. However, as indicated by our results, glutathione-dependent phase II metabolism is distinctly downregulated in this condition, implying a dysfunctional phenotype for effective drug metabolism due to hypoxic induction of β-catenin (Preziosi et al., 2018). Accordingly, cellular response to acetaminophen (APAP) differed widely between cultures, despite specific selection of concentration and duration in order to cause minimal damage to the cells (Jemnitz et al., 2008). APAP metabolism in healthy hepatocytes largely depends on Phase II metabolism via glucuronidation and sulfation (Mazaleuskaya et al., 2015), which inherently depends on cellular energy metabolism due to their co-factors and precursors. Activity of the pericentrally biased (Hinson et al., 2010) Cytochrome P450 (CYP450) system, which results in a cytotoxic metabolite (Mazaleuskaya et al., 2015), on the other hand, does not involve any directly energy-dependent co-factors. Metabolic activity in low flux conditions is thus expected to reduce homeostatic drug detoxification, causing metabolism mainly via the CYP450 system. Indeed, glutathione was found to be consumed at higher rates in low flux cultures during APAP exposure, required for detoxification of CYP450 metabolites (Mazaleuskaya et al., 2015). In comparison, high flux cultures exhibit generally higher transcript levels and co-factor concentrations for *UGT* and *SULT*-related pathways, therefore better mimicking physiological hepatic metabolism (Mazaleuskaya et al., 2015). Accordingly, cytotoxicity was found to increase gradually with lowered ambient oxygen tension in gas-permeable setup, similar to patterns found *in vivo* (Hinson et al., 2010). Ischemic cultures, on the other hand, were damaged at increased rates, possibly leading false estimation of drug-induced hepatocellular injury due to inadequate culturing conditions. Observed morphological changes have been previously reported in galactosamine-induced models of acute liver failure (Wang et al., 2017), indicating the severity of cellular damage due to limited oxygen.

Conclusively, we have shown the complex interactions that need to be considered for accurate hepatic models of cellular drug metabolism. High oxygen supply rates enable cells to maintain homeostatic energy and metabolite levels needed for effective drug detoxification while allowing for oxygen-dependent signaling to induce zonation-like phenotypes. However, in order to avoid the generation of non-specific phenotypes, systematic consideration of signaling factors is necessary. Due to the complexity of the hepatic microarchitecture, apart from biomimetic oxygen supply, this includes media composition in terms of growth factors and hormones, paracrine signaling from non-parenchymal cells (Preziosi et al., 2018), as well as mechanical stress (Lorenz et al., 2018). Limiting oxygen supply, in contrast, triggers a pathophysiological metabolic reprogramming, influencing a wide range of connected cellular functions and reducing the value of these conventional culture systems as physiological *in vitro* models. We have shown that these culture models more closely resemble ischemic or even carcinogenic characteristics. Generally, physiological *in vitro* models are an important first step to effectively and accurately replace *in vivo* models of toxicological testing. If hepatic *in vitro* systems, however, do not model *in vivo* systems accurately, drug-induced injury might be falsely estimated and thus hide possible adverse effects during preclinical development. Oxygen-dependent phenotype induction is a simple way to model specific subpopulations, either zonal or ischemic, in order to improve metabolic understanding and limit short-comings of current hepatocellular systems *in vitro*.

## Data Availability Statement

The datasets generated during the current study are available from the corresponding author on reasonable request.

## Ethics Statement

The animal study was reviewed and approved by University of Tokyo guidelines regarding animal experiments (approval number 2706).

## Author Contributions

BS and YS conceived the study. BS performed the cell culture, biochemical assays, data analysis, and wrote the manuscript. MSh and MD supervised and assisted with individual experiments. MSu performed metabolite quantifications. MN and YS secured funding and assisted with the manuscript. All authors contributed to drafting and revising the manuscript, read and approved the submitted version.

## Conflict of Interest

The authors declare that the research was conducted in the absence of any commercial or financial relationships that could be construed as a potential conflict of interest.

## Supplementary Material

Figure S1 Metabolic patterns induced by differential oxygenation. **(a)** Cellular morphology after 5 days of culture in differential oxygenation (scale bar = 100 μm). **(b)** Measured pericellular oxygen tension in experimental conditions. **(c)** Oxygen consumption limitation in low flux conditions as a fraction of uninhibitedOCR in high flux groups. **(d)** Comparative metabolite enrichment analysis in drug-treated groups. **(e)** Global comparison of mean-centered metabolite values with 50% peak detection rate in both untreated and drug-treated groups.

Figure S2 Time-dependency of zonal phenotype induction. Time-dependent supernatant concentration profiles of functional and metabolic biomarkers. **(a)** Albumin concentration in the culture medium quickly decreases in reduced oxygen conditions, with stabilization occurring after 3 days post-induction. **(b)** Changes in glucose concentration in the culture medium per 24 h show metabolic reprogramming of hepatocyte cultures. Similarly, cultures stabilize their metabolic profile after 3–4 days in culture. **(c)** Lactate production of individual cultures remains largely stable over the duration of the culture with OCR-dependent increase between groups.
