# Supplementary figures and images for: Induction of in vitro Metabolic Zonation in Primary Hepatocytes Requires Both Near-Physiological Oxygen Concentration and Flux
Source: Front Bioeng Biotechnol. 2020 Jun 3;8:524. doi: 10.3389/fbioe.2020.00524 (PMC7325921; doi:10.3389/fbioe.2020.00524)

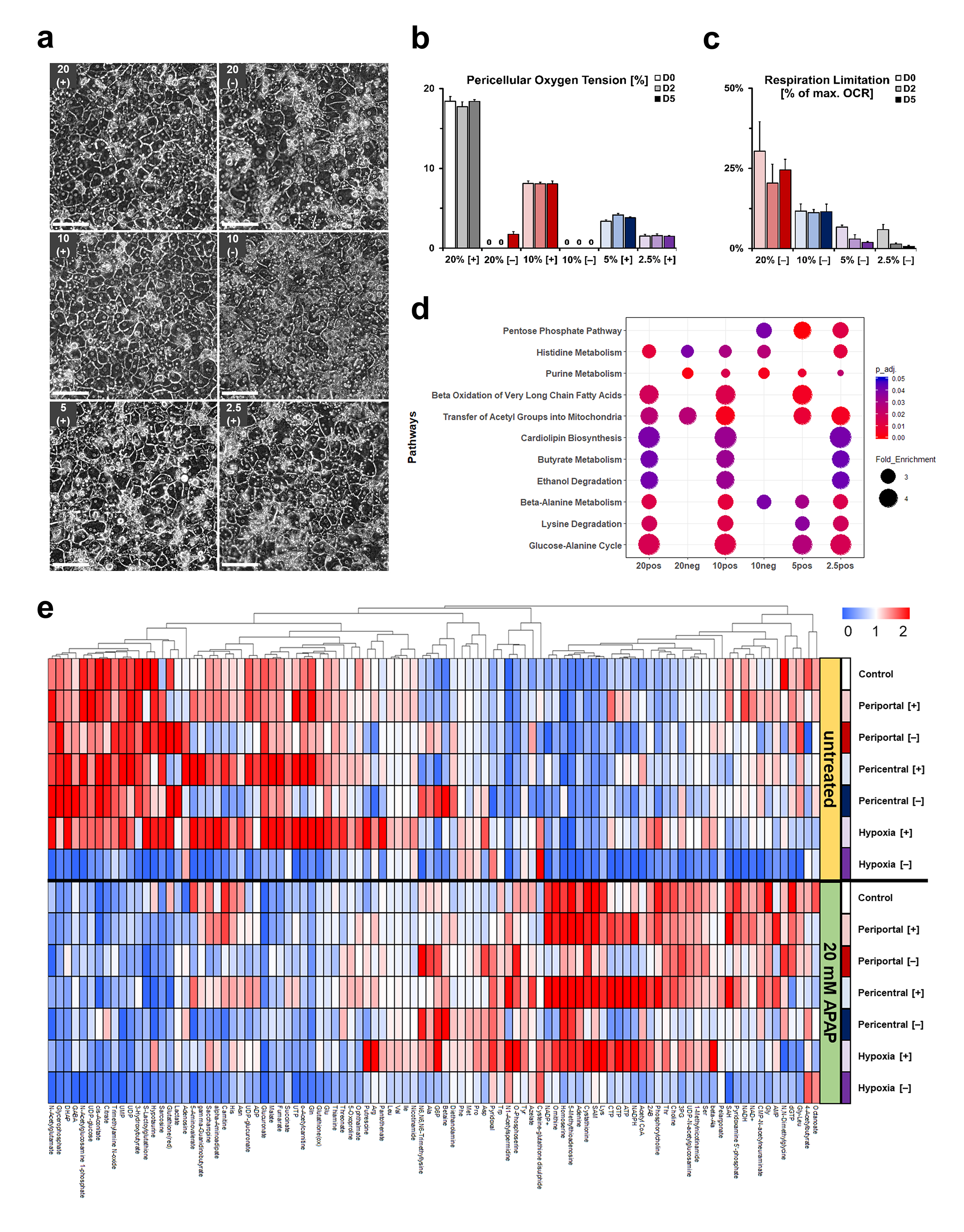

Supplement: Figure S1 — Metabolic patterns induced by differential oxygenation. (a) Cellular morphology after 5 days of culture in differential oxygenation (scale bar = 100 μm). (b) Measured pericellular oxygen tension in experimental conditions. (c) Oxygen consumption limitation in low flux conditions as a fraction of uninhibitedOCR in high flux groups. (d) Comparative metabolite enrichment analysis in drug-treated groups. (e) Global comparison of mean-centered metabolite values with 50% peak detection rate in both untreated and drug-treated groups. [file Image_1.tif]

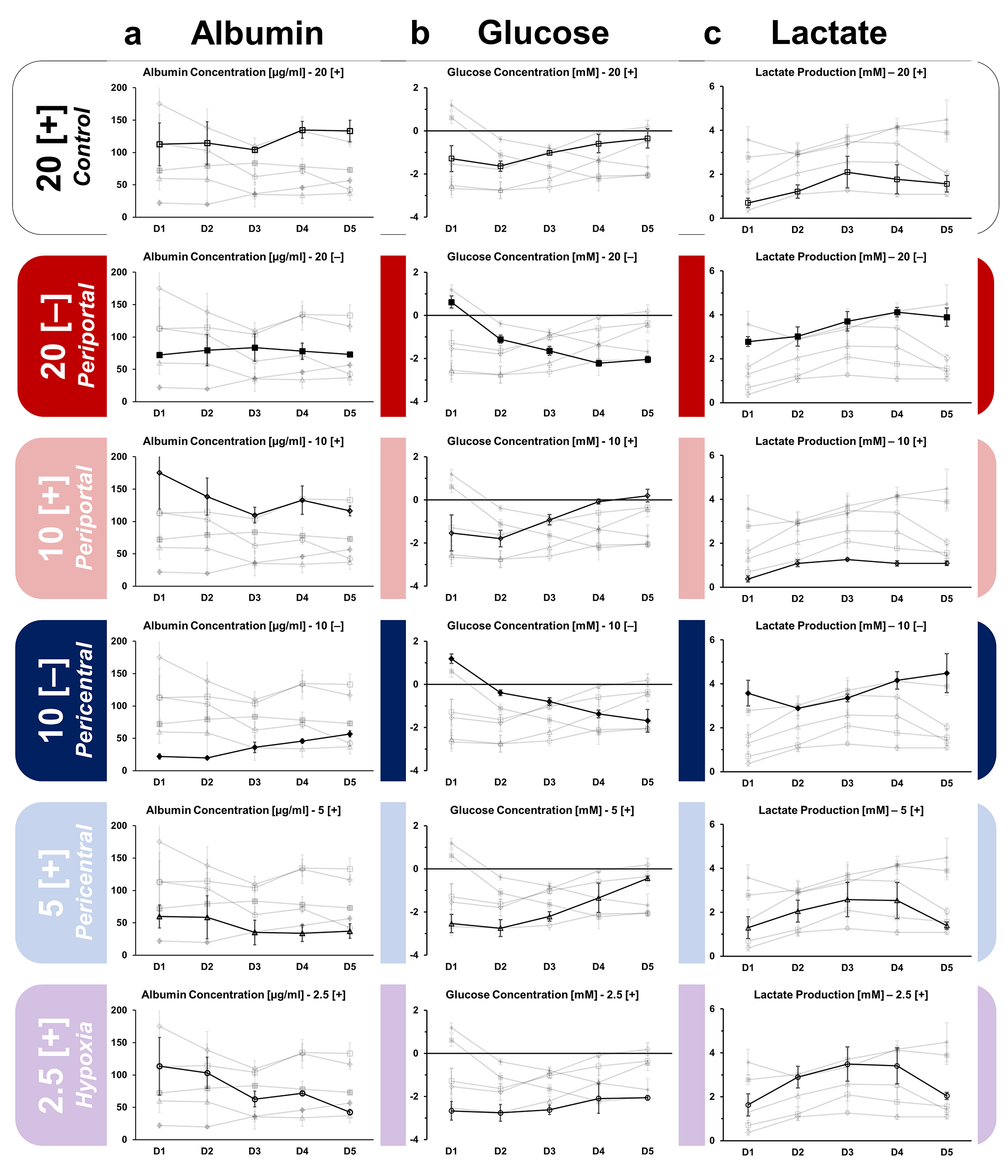

Supplement: Figure S2 — Time-dependency of zonal phenotype induction. Time-dependent supernatant concentration profiles of functional and metabolic biomarkers. (a) Albumin concentration in the culture medium quickly decreases in reduced oxygen conditions, with stabilization occurring after 3 days post-induction. (b) Changes in glucose concentration in the culture medium per 24 h show metabolic reprogramming of hepatocyte cultures. Similarly, cultures stabilize their metabolic profile after 3–4 days in culture. (c) Lactate production of individual cultures remains largely stable over the duration of the culture with OCR-dependent increase between groups. [file Image_2.tif]
